# Reduced suprasellar cistern cerebrospinal fluid motion in patients with Parkinson’s disease revealed by magnetic resonance imaging with dynamic cycling of diffusion weightings

**DOI:** 10.21203/rs.3.rs-3311121/v1

**Published:** 2023-09-07

**Authors:** Gabriela Pierobon Mays, Kilian Hett, Jarrod Eisma, Colin D. McKnight, Jason Elenberger, Alexander K. Song, Ciaran Considine, Caleb Han, Adam Stark, Daniel O. Claassen, Manus J. Donahue

**Affiliations:** Vanderbilt University; Vanderbilt University Medical Center; Vanderbilt University Medical Center; Vanderbilt University Medical Center; Vanderbilt University Medical Center; Vanderbilt University Medical Center; Vanderbilt University Medical Center; Vanderbilt University Medical Center; Vanderbilt University; Vanderbilt University Medical Center; Vanderbilt University Medical Center

**Keywords:** glymphatic, suprasellar cistern, DWI, cerebrospinal fluid, Parkinson’s, α-synuclein, choroid plexus, Parkinson’s disease, cerebrospinal fluid, neurofluid, diffusion weighted imaging, glymphatic, cisterns

## Abstract

**BACKGROUND:**

Parkinson’s disease is characterized by dopamine-responsive symptoms as well as aggregation and accumulation of a-synuclein protofibrils. New diagnostic methods assess a-synuclein aggregation characteristics from cerebrospinal fluid and recent pathophysiologic mechanisms suggest that cerebrospinal fluid circulation disruptions may precipitate a-synuclein retention. Here, we test the hypothesis that cerebrospinal fluid motion at the level of the suprasellar cistern is reduced in Parkinson’s disease relative to healthy participants and this reduction relates to choroid plexus perfusion.

**METHODS:**

Diffusion weighted imaging (spatial resolution=1.8×1.8×4 mm) magnetic resonance imaging with cycling of diffusion weightings (*b*-values=0, 50, 100, 200, 300, 700, and 1000 s/mm^2^) over the approximate kinetic range of suprasellar cistern neurofluid motion was applied at 3-Tesla in Parkinson’s disease (n=27; age=66±6.7 years) and healthy (n=32; age=68±8.9 years) participants. Wilcoxon rank-sum tests were applied to test the primary hypothesis that the decay rate of cerebrospinal fluid signal as a function of *b*-value, which reflects increasing fluid motion, is reduced in persons with versus without Parkinson’s disease and inversely relates to choroid plexus activity assessed from perfusion-weighted magnetic resonance imaging (Spearman rank-order correlation; significance-criteria: *p*<0.05).

**RESULTS:**

Consistent with the primary hypothesis, decay rates were higher in healthy (*D*=0.00328±0.00123mm^2^/s) relative to Parkinson’s disease (*D*=0.00256±0.0094mm^2^/s) participants (*p*=0.016). This finding was preserved after controlling for age and sex. An inverse correlation between choroid plexus perfusion and decay rate (*p*=0.011) was observed in Parkinson’s disease participants.

**CONCLUSIONS:**

Cerebrospinal fluid motion at the level of the suprasellar cistern is often reduced in adults with versus without Parkinson’s disease and this reduction correlates on average with choroid plexus perfusion.

## INTRODUCTION

Parkinson’s disease (PD) is a progressive neurodegenerative disorder with symptoms of cognitive and motor decline, and pathologic aggregation and accumulation of a-synuclein protofibrils^1^. As a-synuclein is distributed throughout the cerebrospinal fluid (CSF), and the topography of a-synuclein clearance and aggregation may contribute to disease propagation in PD, neurofluid motion assessments in PD may be useful to develop new pathophysiologic models of disease progression^2, 3^.

CSF, produced within the choroid plexus complexes, is the primary medium for clearance of waste products and proteins and traverses traditional bulk and recently identified perivascular pathways^4, 5^. Arterial pulsatility is believed to be fundamental to anterograde neurofluid movement, whereas fluid transport along the parasagittal dural spaces and cranial nerves has been proposed to have egress relevance^6, 7^. Major intracranial arteries are bounded by free CSF at the level of the suprasellar cistern, and as such, quantitative imaging of neurofluid motion within the suprasellar cistern may provide one relevant marker of neurofluid circulation secondary to variation in macrovascular arterial pulsatility.

*In vivo* assessments of this motion have been difficult given limited imaging approaches for quantifying neurofluid motion non-invasively *in vivo*. Bulk CSF flow at the level of the cerebral aqueduct can be interrogated using quantitative phase contrast magnetic resonance imaging^7–9^, and perivascular motion can be estimated qualitatively from *T*_2_-weighted MRI or *T*_1_-weighted MRI before and after intrathecal contrast agent administration^10^. However, such imaging of perivascular motion generally does not provide quantitative measures of motion and additionally suffers from the caveats that intrathecally administered contrast is contraindicated for research studies in most centers and may not reflect *in vivo* neurofluid kinetics.

To begin to address this limitation, we implemented a novel diffusion weighted imaging (DWI) magnetic resonance imaging (MRI) protocol and analysis approach, which incorporates dynamic diffusion weighting cycling over the approximate kinetic range of CSF motion within the suprasellar cistern. This approach, which has previously been proposed in healthy adults using categorical scoring of signal attenuation for increasing diffusion weighting^11^, is applied here to (i) demonstrate sensitivity to regions with known differences in neurofluid motion and (ii) quantify relationships between neurofluid motion and choroid plexus perfusion in older adults with and without PD. The hypothesis to be investigated is that neurofluid motion within the suprasellar cistern of patients with PD is reduced relative to age-matched controls. A secondary hypothesis is that markers of CSF production activity increase in the presence of reduced neurofluid movement within the suprasellar cistern, which would be consistent with additional CSF production activity required to clear CSF along less compliant perivascular pathways as has been suggested in separate vascular disorders^12^. Findings are discussed in the context of the growing literature on neurofluid circulation and neurodegeneration.

## METHODS

### Demographics

Adult participants with and without Movement Disorder Society clinical criteria for PD^13^ with no other history of neurological disease were recruited and provided informed consent for this prospective Institutional Review Board (IRB)-approved study. Disease duration as well as the Unified Parkinson’s disease rating scale (UPDRS^14^) were both recorded in all participants. Exclusion criteria were: independent neurological or psychiatric condition including but not limited to Alzheimer’s disease, multiple sclerosis, prior overt stroke, schizophrenia, or bipolar disorder; flow-limiting cervical or intracranial stenosis (i.e., stenosis > 70%) of the internal carotid arteries, vertebral or basilar arteries, or first segment of the anterior, middle, or posterior cerebral arteries; independent condition expected to lead to death within one year; and any contraindication to 3-Tesla MRI. Presence of non-specific white matter lesions was not an exclusion criterion, as these lesions become more prevalent with aging, and we sought our cohort to be generalizable and representative. Parkinsonism medications (e.g., dopamine agonist and dopamine replacement therapies) were withheld for 16-hours prior to scanning. Exclusion criteria were identical for healthy control participants and additionally expanded to include positive mental health negative for neuro-psychiatric conditions and no complaints of prior cognitive issues.

### Non-imaging assessments

PD and healthy control participants underwent cognitive screening with the Montreal Cognitive Assessment (MoCA)^15^ and brief neuropsychological test examination with the Repeatable Battery for the Assessment of Neuropsychological Status (RBANS)^16^. PD participants were additionally screened by a board-certified neurologist (DOC) to ensure PD criteria were met^14^.

### Experiment

Participants were scanned at 3-Tesla (Philips Healthcare, Best, The Netherlands) using body coil radiofrequency transmission and SENSE phased-array 32 channel reception. As it has been shown that CSF production activity is coupled to circadian rhythms^17^, we imaged consistently between 7:00 AM and 11:00 AM.

To characterize tissue and vascular health, a standard non-contrast head protocol was applied: (i) 3D *T*_1_-weighted magnetization-prepared-rapid-gradient-echo (TR = 8.1 ms; TE = 3.7 ms; field-of-view = 256×180×150 mm; slices = 150; spatial resolution = 1.0×1.0×1.0 mm; duration = 4:32), (ii) 2D *T*_2_-weighted fluid-attenuated-inversion-recovery (FLAIR) turbo-spin-echo (TR = 11s; TE = 120 ms; TI = 2,800 ms; field-of-view = 230×184×144 mm; slices = 29; spatial resolution = 0.57×0.57×4.0 mm; duration = 1:39), (iii) 3D *T*_2_-weighted turbo-spin-echo (TR = 2,500 ms; TE = 331 ms; field-of-view = 250×250×189 mm; slices = 242; spatial resolution = 0.78×0.78×0.78 mm; duration = 4:08), (iv) 2D diffusion-weighted spin-echo with single-shot echo-planar imaging readout (TR = 2,926 ms; TE = 83 ms; field-of-view = 229×229×139 mm; slices = 28; spatial resolution = 1.8×1.8×4.0mm; *b*-value = 1000 s/mm^2^; duration = 0:58), and (v) 3D time-of-flight magnetic resonance angiography (TR = 23 ms; TE = 3.5 ms; field-of-view = 200×200×84 mm; slices = 120; spatial resolution = 0.39×0.39×0.70 mm; duration = 4:07). These images and angiograms were used to ensure inclusion criteria were met.

For functional assessment of suprasellar cistern fluid motion and choroid plexus perfusion, multi-shell DWI and pseudo-continuous arterial spin labeling (pCASL), respectively, were applied in sequence. 2D echo-planar-imaging DWI with common field-of-view = 230×230×139 mm, diffusion directions = 6, slices = 28, spatial resolution = 1.8×1.8×4 mm, TE = 61 ms, and TR = 2,539 ms were acquired with cycling of seven pseudo-randomized *b*-values (*b*-values = 0, 50, 100, 200, 300, 700, and 1000 s/mm^2^). To evaluate choroid plexus perfusion, a recently-reported pCASL approach tested for reproducibility and sensitivity^8, 12^ to choroid plexus perfusion was used with TR = 4,550 ms, TE = 11 ms, post-label delay = 2,000 ms and with labeling (0.5ms Hanning-windowed pulses; pulse-train duration = 1800 ms) placed proximal to anterior and posterior choroidal arteries (which supply the choroid plexus), approximately 100 mm proximal to the corpus callosum. A TR = 15s acquisition with identical geometry and spin labeling removed was acquired for equilibrium magnetization (M_0_) determination.

### Analysis

Anatomical imaging and angiography scans were reviewed by a board-certified radiologist (CDM; experience = 9 years) to ensure that no exclusion criteria were met. Clinical history was reviewed by a board-certified neurologist (DOC; experience = 16 years) to ensure participants met healthy control or PD criteria. A board-certified neuropsychologist (CMC; experience = 5 years) reviewed all cognitive screening and neuropsychological test data to ensure stated inclusion criteria regarding cognition were met.

Choroid plexus segmentations were generated from a previously published^8^ fully convolutional neural network with 3-D U-Net architecture utilizing co-registered *T*_1_-weighted and *T*_2_-weighted FLAIR images in 1 mm isotropic MNI152 space. All registrations were performed using the Advanced Normalization Tools (ANTs) nonlinear registration tools^18^. Choroid plexus perfusion was quantified within the atria of the lateral ventricles following previously reported procedures^8^. Briefly, the source pCASL control and label images were surround subtracted, slice delay corrected, M_0_-normalized, averaged, and fit to a general kinetic model^19^ using the following parameters: blood-brain partition coefficient (λ) = 0.9 mL blood/g brain, labeling efficiency from the dual-background suppressed sequence (α) = 0.8, and the 3T *T*_1,blood_ = 1,624 ms. The processed perfusion maps were co-registered to the *T*_1_-weighted images using the ANTs linear registration tools^18^, and the choroid plexus segmentation from the machine learning model was applied to the perfusion maps to calculate the mean perfusion values in the choroid plexus of the lateral ventricles. A minimum threshold of 10 mL/100 g/min was used for perfusion quantification to ensure that all values were above the noise floor.

Maps of neurofluid motion within the suprasellar cistern were calculated from the multi-shell DWI scan. Images were corrected for bulk head motion^18^ and an exponential decay model was fit voxel-wise to the maps:

[1] $$S\left(b\right)=S_{0}\;exp(-b\cdot D)$$

where S is the signal as a function of *b*-value, D is the free diffusion coefficient for water, and $S_{0}$ is signal in the absence of diffusion-sensitizing gradients, and *b* is the *b*-value=(gGd^2^D for the narrow pulse approximation with gradient amplitude *G*, the diffusion time *Δ*, and gradient pulse width, *δ*^20^. Note that in this fitting procedure, which is analogous to the calculation of the apparent diffusion coefficient (ADC), larger decay rates (D) reflect faster decay across the *b*-value range and as such higher neurofluid motion. After model fitting, resulting neurofluid motion maps were up-sampled to 1 mm isotropic resolution, and spatially normalized to the MNI space using symmetric normalization (SyN)^18^. Spatial transformations utilized the b = 50 s/mm^2^ image as a template. The region comprising the suprasellar cistern was manually identified in template space by a board-certified radiologist (CDM; experience = 9 years) and decay values were averaged within this region for each participant. In addition, gray matter, white matter, and CSF masks were used to quantify the decay rates in each tissue region.

### Statistical considerations and hypothesis testing

Descriptive statistics, including means and standard deviations of continuous imaging parameters, as well as percentages and frequencies of categorical parameters, were calculated. A Wilcoxon rank sum test was applied to evaluate differences in continuous measures, whereas a chi-squared test was applied to evaluate differences in categorical measures. First, to ensure that the multi-shell DWI protocol was sensitive to known regions of increased neurofluid movement, voxel-wise rate (D) values in CSF, gray matter, and white matter were contrasted across all participants to test the hypothesis that motion (mm^2^/s) was highest in CSF compared to gray matter and white matter. Summary statistics which describe normative values in each region for PD and control participants were recorded. Second, to test the overarching hypothesis that suprasellar cistern neurofluid motion was reduced in PD relative to control participants, a Wilcoxon rank sum test was applied between mean rates in the suprasellar cistern for each cohort; separate linear regression analyses which modeled the dependence of the decay rate on (i) cohort, (ii) age, and (iii) sex were performed. Finally, to understand whether reduced suprasellar cistern motion was related to elevated choroid plexus activity, Spearman rank-order correlation test was applied using the decay rate as the dependent variable and choroid plexus perfusion as the independent variable, separately for healthy and PD participants. As an exploratory analysis, separate regression analyses were performed to evaluate any dependence of (i) UPDRS (motor function) or (ii) MoCA (cognitive function) on the independent variable of decay rates within the suprasellar cistern region in PD participants. For all comparisons, significance was defined as two-sided *p* < 0.05.

## DATA SHARING

Source imaging and demographic data will be made available to investigators with appropriate Collaborative of Institutional Training Initiative *Responsible Conduct of Human Research* and *Good Clinical Practice* training upon request.

## RESULTS

The participants enrolled are summarized in Table 1, which comprised 59 participants, including 27 with PD (age = 66 ± 6.7 years) and 32 healthy adults (age = 68 ± 8.9 years). Participants between groups were matched for age (*p* = 0.465) and no participants had evidence of vasculopathy or met neurological or radiological exclusion criteria. One healthy control with atypical cognitive screen (MoCA < 24)^21^ was determined to demonstrate clinically significant performance deficits on neuropsychological test examination (1 + score < 2 standard deviations, or, 2 + scores < 1.5 standard deviations)^22^. Five healthy controls with atypical cognitive screens (MoCA = 22–23) were determined to demonstrate performance on neuropsychological test examination within normal limits, and thus were included in subsequent analyses.

**Figure 1:**
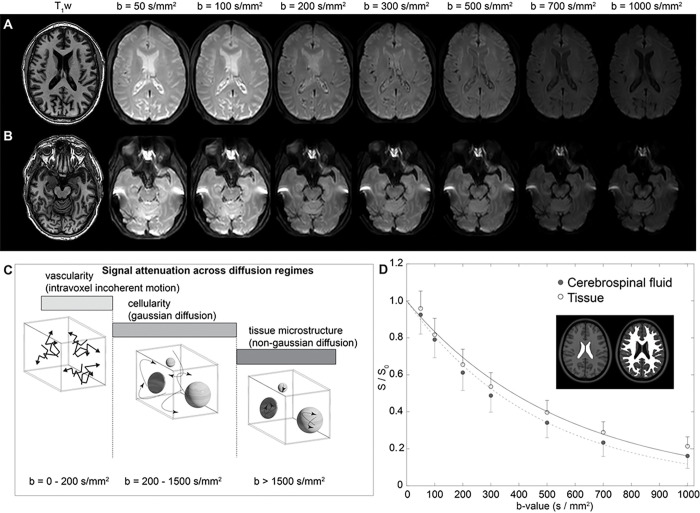
Diffusion weighted imaging (DWI) with dynamic cycling of low-to-intermediate *b*-values. (A) Two slices at the level of the lateral ventricles (A) and suprasellar cistern (B) for a representative participant (age=77 years; sex=male) are shown. (C) The approximate regime of physiological sensitivity for increasing *b*-values, whereby low *b*-values < 200 s/mm^2^ have known sensitivity to vascular structures and intravoxel incoherent motion, intermediate *b*-values of approxiamtely 200 – 1500 s/mm^2^ are sensitive to cellularity in the regime of gaussian diffusion, and high *b*-values above 1500 s/mm^2^ are most sensitive to non-gaussian diffusion and tissue microstructure assessments. (D) Example decay curves as a function of low-to-intermediate *b*-value in the transition range of intravoxel incoherent motion and gaussian diffusion demonstrate differences in cerebrospinal fluid and tissue. Error bars across the region shown in the insert are depicted as one-sided for clarity.

Figure 1A shows acquired representative DWI in a participant as a function of increasing *b*-value, whereby faster flowing fluid is suppressed in all *b*-values and slower flowing fluid is suppressed in larger *b*-values only. Corresponding decay maps (mm^2^/s), calculated using the prescribed low-to-intermediate *b*-values of 0–1000 s/mm^2^, in the native space of the participant are shown in Fig. 1B. Figure 1C summarizes the range of diffusion relative to different water motion, including intravoxel incoherent motion, gaussian diffusion, and non-gaussian diffusion, and the relevant approximate diffusion weightings for each regime. Figure 1D shows accelerated signal decay at the level of the CSF relative to tissue for the low *b*-values used here. It should be noted that since parenchymal motion in this range may be attributable to capillary flow or neurofluid flow, we focused our analysis on neurofluid motion within the suprasellar cistern.

**Figure 2:**
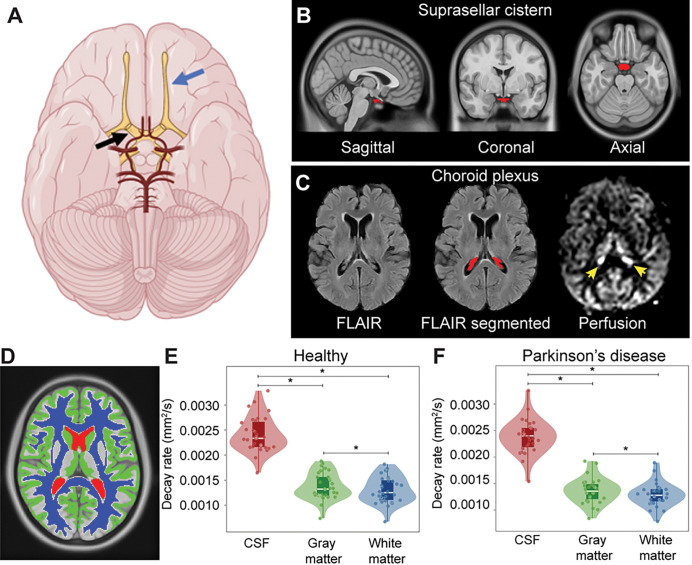
Localization and segmentation of imaging regions. (A) Suprasellar cistern location, which co-localizes with major cranial nerves including the olfactor (blue arrow) and optic (black arrow), along with circle of Willis (red vasculature). (B) Suprasellar cistern region of interest used in all participants overlaid on the standard 1 mm brain atlas. (C) Choroid plexus at the level of the lateral ventricles visible on FLuid Attenuated Inverstion Recovery (FLAIR) MRI, along with example of segmentation; the perfusion map is shown to right, which highlights (yellow arrows) the high choroid plexus perfusion signal which is comparable to gray matter perfusion signal. Given the high variability in choroid plexus anatomy, structures were segmented in native space for each participant using previously-reported machine learning routines (see Methods). (D) Example of tissue segmentation used to confirm abilities to evaluate differences in neurofluid and tissue motion. (E-F) Separately in each group, decay rates are significantly elevated in cerebrospinal fluid (CSF) relative to other tissue types (*p*<0.001), a well as in gray matter relative to white matter (*p*<0.001). The plots show boxplots overlaid on violin plots, with all participant data plotted as separate data points for completeness. See Table 1 for quantitative values. * *p*<0.001.

Figure 2 depicts the common suprasellar cistern region of interest, both in terms of the location relative to arteries of the circle of Willis and cranial nerves. The common suprasellar cistern region of interest evaluated in all participants is shown in Fig. 2B and an example of the deep learning algorithm applied to isolate the choroid plexus at the level of the lateral ventricles is shown in Fig. 2C. Note that given inter-subject variability in choroid plexus anatomy, this segmentation was performed for each participant. To confirm that the method could detect differences in regions with known fluid differences, Fig. 2D shows the gray matter, white matter, and CSF segmentation, along with violin plots of decay rates in Fig. 2E (Eq. 1). Decay rates were significantly elevated (p < 0.001) in CSF relative to other tissues, as well as in gray matter (p < 0.001) compared to white matter. This finding was observed both in the PD and healthy participants.

**Figure 3:**
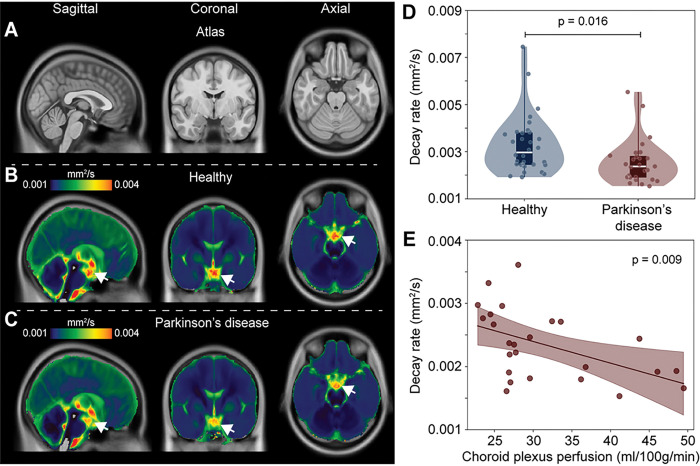
Group-averaged maps of decay rates in healthy participants and participants with Parkinson’s disease (PD). Reduced supracellar flow is observed in PD relative to healthy participants (A-C). (D) Violin plots showing the distribution of suprasellar cistern decay rates in healthy vs. PD participants, suggesting reduced suprasellar cistern neurofluid motion in PD (*p*=0.016). The plots show boxplots overlaid on violin plots, with all participant data plotted as separate data points for completeness. (E) In PD participants only, reduced decay rates, indicative of slower neurofluid flow, are associated with higher choroid plexus perfusion values.

Figure 3 shows group level decay rate maps, separately for the healthy adults and PD participants. When group level values were considered in the suprasellar cistern, the decay rates were found to be significantly higher in healthy (D = 0.00328 ± 0.00123 mm^2^/s) relative to PD (D = 0.00256 ± 0.0094 mm^2^/s) participants (*p* = 0.016), consistent with the primary study hypothesis. There was no relationship observed for the decay rate and age (*p* = 0.342) or sex (*p* = 0.604) among PD and healthy participants on linear regression. Figure 3 also summarizes the distribution of decay rates across cohorts and against choroid plexus perfusion. When the total cohort was considered, there was no relationship that was observed between the decay rate and choroid plexus perfusion (*p* = 0.369). However, an inverse correlation between choroid plexus perfusion and decay rate (Spearman’s r = −0.51; *p* = 0.011) was observed when only PD participants were considered. All analyses met criteria with and without the single healthy control that met stated criterial for mild cognitive impairment.

Regression analyses yielded a trend (Spearman’s r = 0.264; *p* = 0.183) between UPDRS score and suprasellar cistern decay rate, consistent with higher levels of motor dysfunction being associated with reduced suprasellar fluid motion, however, this did not meet stated significance criteria. No trend for a relationship was observed with the MoCA score (Spearman’s r=−0.008; *p* = 0.968).

**Figure 4:**
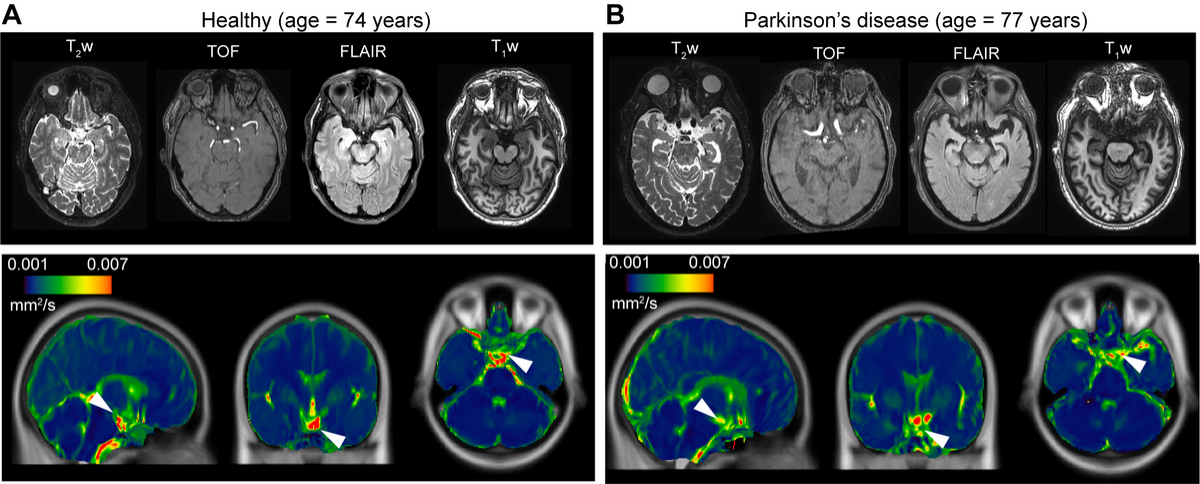
Case examples. Case example of an age- and sex-matched healthy (A) and Parkinson’s disease (PD) (B) participant showing anatomical imaging at the level of the suprasellar cistern. For the same two participants, orthogonal depictions of the atlas and the the quantified decay rate maps are shown, scaled identically, which demonstrate reduced suprasellar cistern motion in the PD relative to healthy participant (white arrows: suprasellar cistern on quantified maps).

Case examples are shown in Fig. 4, whereby differences in suprasellar cistern fluid motion are identifiable on quantified maps.

## DISCUSSION

Diffusion weighted imaging (DWI) MRI with dynamic cycling of diffusion weightings over the approximate kinetic regime of suprasellar cistern CSF was applied in adults with PD and age-matched healthy controls. Significantly attenuated motion was observed in the suprasellar cistern of PD relative to healthy participants and the extent of motion attenuation was associated with increased choroid plexus perfusion in PD participants. The methodology proposed, which is similar to an apparent diffusion constant calculation from low *b*-value DWI MRI, can be implemented on clinical scanners and could represent an additional non-invasive tool for assessment of neurofluid kinetics in the setting of neurodegeneration.

### Expansion of methodologies to assess fluid movement non-invasively in vivo

Modeling diffusion signal decay as a function of *b*-value in principle provides quantitative kinetic information and may present a candidate non-tracer technology for quantifying neurofluid movement non-invasively *in vivo*. This methodology should be considered in light of other MRI-based assessments of quantitative fluid movement. Most commonly, phase contrast magnetic resonance angiography, generally with velocity encoding gradients of 50–100 cm/s or higher can be used to quantify peak systolic arterial flow velocities in the major cervical vessels (velocity = 75–125 cm/s)^23^ or intracranial vessels of the circle of Willis (70–160 cm/s)^24^. In major venous structures, peak systolic velocity is lower and within a healthy range of 10–35 cm/s for most major venous structures^25^. Similar phase contrast methods can be applied at the level of the cerebral aqueduct to assess CSF flow *en route* from the third to fourth ventricle, as peak CSF flow velocity is frequently reported as 10–20 cm/s^7–9^. However, these phase contrast approaches are generally not practical for assessing CSF movement in other structures given much slower and non-laminar flow. Under these physiological conditions, sufficiently low velocity encoding gradients are beyond the hardware limits of clinical scanners. Blood arrival to tissue, and even CSF flow^26^ and peripheral lymphatic flow^27^, have been assessed using principles of spin labeling with multiple delay times. However, the duration of the generated magnetic label is governed by the T_1_ relaxation time of the water spins in the separate fluids, which, at 3-Tesla, is approximately 1,624ms for blood water^28^, 1,442ms for CSF^29^, and 610ms for lymphatic fluid^27^. Therefore, principles of spin labeling can only be applied to assess fluid movement over short distances or when velocity is high.

This work utilizes principles of diffusion and the slower and more isotropic fluid motion in the suprasellar cistern to estimate neurofluid kinetics. The theory of estimating diffusion properties from different levels of diffusion weightings has been well characterized^30^ and the approach proposed here is similar to a calculation of the apparent diffusion coefficient, albeit over a range of diffusion weightings relevant to CSF motion. Recent work has applied DWI at different *b*-values to qualitatively estimate neurofluid motion from categorical scoring of the rate of decay in ten healthy adults (aged = 25–58 years) in posterior fossa, suprasellar cistern, and Sylvian vallecula compared to the lateral ventricle and frontal and parietal CSF spaces^11^. This work also provided support for greater CSF fluid motion in the third ventricles in younger versus older participants, however, the method required radiological scoring and non-continuous, quantitative assessment was not proposed. Our work extends the approach to enable quantitative, continuous assessments of the decay rate.

### Neurofluid motion within the suprasellar cistern

The suprasellar cistern is located at the level of the circle of Willis, superior to the sella turcica, anterior and inferior to the hypothalamus, and bordered by the uncus of the temporal lobes. Importantly, the suprasellar cistern is filled with freely circulating CSF and additionally contains the optic chiasm, the infundibular stalk, and the cerebrovascular circle of Willis.

Growing evidence suggests that pulsatility of the intracranial vasculature is fundamental to neurofluid motion^31, 32^. Compliance of the cerebrovasculature and healthy pulsatility assists with movement of neurofluids within the cerebrum, both at the level of the parenchyma and in large CSF-filled cisterns. In support of this, patients with bilateral intracranial vasculopathy have been observed to have elevated perfusion of the choroid plexus, which may be required to upregulate CSF production activity in the presence of reduced pulsatility of arteries secondary to vasculopathy^33^. It has also been shown that choroid plexus perfusion reduces after successful angiogenesis-inducing indirect surgical revascularization in these patients^33^, and also, that the choroid plexus activity may be reduced in the presence of hyper-vascularity and sickle cell anemia, where increases in cerebral blood volume are required to compensate for reduced blood oxygen content from anemia^12^.

The observation that neurofluid motion has high inter-subject variability in the cisterns has previously been shown in the intracranial vessel wall imaging literature^34^, whereby delay-alternating-nutation-with-tailored-excitation fluid suppression modules^35^ were utilized to null basal cistern CSF signal in a velocity-dependent manner, which when effective increases conspicuity of the intracranial vessel walls. However, the degree of fluid attenuation has been reported to be highly participant-dependent, a finding attributed to high variability of inter-subject neurofluid motion within the basal cisterns^34^. This work extends these findings to demonstrate that neurofluid motion within the suprasellar cistern varies between PD and age-matched healthy adults, and importantly, this variation is inversely related to the perfusion of the choroid plexus in PD participants. These findings taken together provide support for the choroid plexus upregulating activity in the presence of reductions in intracranial neurofluid motion and arterial pulsatility.

### Neurofluid aberrations in Parkinson’s disease

There is a growing literature reporting aberrant neurofluid circulation in PD, as well as other neurodegenerative proteinopathies^36^. Specifically with regards to PD, dynamic contrast-enhanced magnetic resonance imaging has been applied to assess meningeal lymphatic flow in cognitively normal controls and patients with idiopathic PD or atypical Parkinsonian disorders, and it was observed that patients with idiopathic PD exhibited significantly reduced flow through the meningeal lymphatic vessels along the superior sagittal sinus and sigmoid sinus, as well as a notable delay in deep cervical lymph node perfusion, compared to patients with atypical Parkinsonian disorders^37^. These findings suggest that differences in neurofluid circulation may partly underlie differences between idiopathic and atypical Parkinsonian disorders. Furthermore, mice injected with α-synuclein preformed fibrils demonstrated delayed meningeal lymphatic drainage, loss of tight junctions among meningeal lymphatic endothelial cells and increased inflammation of the meninges^37^.

Prior work using principles of diffusion to estimate water motion along perivascular spaces has provided evidence in support of reduced water motion along perivascular spaces in older adults with PD relative to essential tremor^2^. This finding was tentatively attributed to PD being a proteinopathy and that neurofluid movement aberrations may contribute to the retention of a-synuclein. This finding is partly consistent with other work: aquaporin-4 (AQP4) channels are likely essential for effective transport of fluid between perivascular and interstitial spaces, and separate work has shown that a-synuclein deposition negatively correlates with AQP4 expression in patients with PD^38^. Additionally, Fang et al.^39^ evaluated AQP4 polymorphisms in PD together with Positron Emission Tomography measures of beta-amyloid burden, sleep behavior, and CSF biomarkers and observed that AQP4 rs162009 may be a biomarker of cognitive decline in PD, possibly attributable to its role in changing neurofluid circulation.

Finally, we observed no relationship between cognition, assessed with MoCA, and the imaging measures, however, this is not surprising given that most participants enrolled in this work had no or only very mild cognitive impairment. For motor impairment, assessed with UPDRS, we observed a trend for a relationship between increased motor dysfunction and slower neurofluid motion within the suprasellar cistern, however, the study was not powered to evaluate this relationship rigorously, which would require approximately 60 patients and likely a multiple regression analysis with additional control for other relevant covariates such as age, sex, and disease duration. As the proposed method can be readily applied in 5–10 min, future work that assesses how neurofluid motion within the cisterns relates to symptomatology, together with other established markers of disease severity, may be useful.

### Limitations

First, the spatial resolution of typical DWI scans at the clinical field strengths of 3 Tesla is 8–27 mm^3^, which is at or larger than the size of many structures relevant to perivascular flow. Therefore, we focused our study on the suprasellar cistern, which has a volume more than 300-fold larger than the spatial resolution of the voxel. Since the suprasellar cistern contains the major arteries of the circle of Willis, and is comprised of freely circulating CSF, it represents a logical location for assessment of neurofluid motion. However, we did not attempt to interrogate smaller structures with emerging relevance to glymphatic flow (e.g., cribriform plate, cranial nerves, or parasagittal dural spaces) nor perform spatial analysis of variance analyses given the sizes of these structures relative to the spatial resolution of the DWI acquisition. Second, given limited information available on kinetic neurofluid movement within the suprasellar cistern, it remains unclear which velocity or diffusion regime is most relevant. Work by others in 50 healthy adults and 50 patients with ventricular dilation demonstrated that at the *b*-value of 500 s/mm^2^, signal voids in 3rd and 4th ventricles, the cerebral sulci and the Sylvian fissure were observed, thereby providing evidence for this range being reasonable for assessing CSF flow^40^. Therefore, we applied diffusion weightings 0–1000 s/mm^2^ and characterized the signal decay quantitatively. Future work will benefit from further defining the precise range of diffusion weightings that are most relevant.

In conclusion, diffusion weighted imaging with dynamic cycling of *b*-values was applied to provide evidence supporting reduced CSF motion within the suprasellar cistern in participants with versus without Parkinson’s disease. The extent of attenuation of fluid movement corresponded with choroid plexus hyperemia in PD participants. These findings suggest that low *b*-value DWI may provide a new imaging tool to interrogate neurofluid motion in the growing number of applications where neurofluid circulation dysfunction is implicated.

## Figures and Tables

**Table 1:** Participant demographics and measures of suprasellar cistern motion and choroid plexus perfusion.

	Healthy	Parkinson’s disease	*p*-value
N	32	27	
Age (years)	67.6 ± 9.0	66.0 ± 6.8	0.465
Unified Parkinson’s Disease Rating Scale (UPDRS)	n / a	32.4 ± 12.5	-
Montreal Cognitive Assessment (MoCA)	26.5 ± 2.8	24.3 ± 3.9	0.026*
Vasculopathy present (percent)	0 ± 0	0 ± 0	1.000
Ventricle decay rate, D (mm^2^/s)	0.00241 ± 0.00035	0.00239 ± 0.00033	0.792
Total gray matter decay rate, D (mm^2^/s)	0.00138 ± 0.00026	0.00136 ± 0.00024	0.832
Total white matter decay rate, D (mm^2^/s)	0.00131 ± 0.00026	0.00129 ± 0.00023	0.749
Suprasellar cistern decay rate, D (mm^2^/s)	0.00328 ± 0.00123	0.00256 ± 0.00094	0.016*
Choroid plexus perfusion (ml/100g/min)	31.3 ± 5.6	31.5 ± 7.9	0.930

n / a: not applicable.

* significant at stated criteria two-sided p < 0.05.

## Availability of data and materials:

Source imaging and demographic data will be made available to investigators with appropriate Collaborative of Institutional Training Initiative Responsible Conduct of Human Research and Good Clinical Practice training upon request.

## Abbreviations

ADC: apparent diffusion coefficient
ANTs: Advanced Normalization Tools
AQP4: aaquaporin-4
CSF: cerebrospinal fluid
DWI: diffusion weighted imaging
FLAIR: FLuid Attenuated Inverstion Recovery
IRB: Institutional Review Board
MoCA: Montreal Cognitive Assessment
MRI: magnetic resonance imaging
pCASL: pseudocontinuous arterial spin labeling
PD: Parkinson’s disease
RBANS: Repeatable Battery for the Assessment of Neuropsychological Status
TE: echo time
TR: repetition time
UPDRS: Unified Parkinson’s Disease Rating Scale
